# “Real-life” continuous flash suppression (CFS)-CFS with real-world objects using augmented reality goggles

**DOI:** 10.3758/s13428-018-1162-0

**Published:** 2018-11-14

**Authors:** Uri Korisky, Rony Hirschhorn, Liad Mudrik

**Affiliations:** 1 School of Psychological Sciences, Tel-Aviv University, Ramat Aviv, POB 39040, 69978 Tel Aviv, Israel; 2 Sagol School for Neuroscience, Tel-Aviv University, Ramat Aviv, POB 39040, 69978 Tel Aviv, Israel

**Keywords:** Continuous flash suppression, Consciousness, Unconscious processing, Real objects, Augmented reality

## Abstract

Continuous flash suppression (CFS) is a popular method for suppressing visual stimuli from awareness for relatively long periods. Thus far, this method has only been used for suppressing two-dimensional images presented on screen. We present a novel variant of CFS, termed “real-life” CFS, in which a portion of the actual immediate surroundings of an observer—including three-dimensional, real-life objects—can be rendered unconscious. Our method uses augmented reality goggles to present subjects with CFS masks to the dominant eye, leaving the nondominant eye exposed to the real world. In three experiments we demonstrated that real objects can indeed be suppressed from awareness for several seconds, on average, and that the suppression duration is comparable to that obtained using classic, on-screen CFS. As supplementary information, we further provide an example of experimental code that can be modified for future studies. This technique opens the way to new questions in the study of consciousness and its functions.

**Electronic supplementary material:**

The online version of this article (10.3758/s13428-018-1162-0) contains supplementary material, which is available to authorized users.

In the last decades, the field of consciousness studies has experienced substantial growth and development (Koch, Massimini, Boly, & Tononi, 2016; Kouider & Dehaene, 2007; Lau, 2017). Once considered to be a question outside the scope of scientific investigation, the neural correlates and possible functions of conscious awareness are now studied widely. Accordingly, a myriad of new, elegant techniques to suppress stimuli have emerged (Kim & Blake, 2005; Lin & He, 2009; for reviews and comparisons, see the special issue by Dubois & Faivre, 2014), all leading to subjects’ inability to report seeing a stimulus that has been presented to them.

One of the most popular methods for rendering stimuli invisible is *continuous flash suppression* (CFS; Tsuchiya & Koch, 2005), which is a variant of binocular rivalry (e.g., Blake & Logothetis, 2002). In CFS, a dynamic pattern of shapes (often referred to as “Mondrians”) is presented to the subject’s dominant eye, usually alternating at a rate of around 10 Hz. At the same time, a target stimulus is displayed to the nondominant eye. As a result, the target stimulus is suppressed from awareness, and the subject perceives only the dynamic pattern of shapes for relatively long durations, sometimes as long as tens of seconds (see again Tsuchiya & Koch, 2005).

CFS has been widely utilized to study the unconscious processing of information; since it was introduced in the field, it has been used in more than 250 studies, probing different types of unconscious processing (for reviews, see Gayet, Van der Stigchel, & Paffen, 2014; Yang, Brascamp, Kang, & Blake, 2014). The mechanisms of CFS are still unclear, however; for example, it is not yet known whether the suppression achieved with this method is driven by low-level processes controlling the gain of visual information arriving from each eye, or by higher-level processes (Watanabe et al., 2011; Yuval-Greenberg & Heeger, 2013). Similarly, it is still debated whether CFS differentially activates the ventral and dorsal streams (Almeida, Mahon, & Caramazza, 2010; Fang & He, 2005; Rothkirch & Hesselmann, 2018; Sterzer, Stein, Ludwig, Rothkirch, & Hesselmann, 2014). Perhaps not surprisingly, then, the results obtained with CFS have also somewhat conflicted: Some studies have shown that CFS abolishes well-known psychophysical phenomena (e.g., Harris, Schwarzkopf, Song, Bahrami, & Rees, 2011; Laycock, Sherman, Sperandio, & Chouinard, 2017). Others have reported high-level semantic processing of the suppressed stimuli (e.g., Costello, Jiang, Baartman, McGlennen, & He, 2009; Sklar et al., 2012; but see, among others, Moors, Hesselmann, Wagemans, & van Ee, 2017a; Shanks, 2017). Thus, the literature seems to be divided as to the scope of the unconscious processing of information under CFS. Notwithstanding these controversies, the methodology itself has proved to be highly fruitful in consciousness studies and has opened the gate to new lines of research and new types of questions that have necessitated longer-duration stimuli (e.g., studying temporal intergration (Faivre & Koch, 2014) or causality (Moors, Wagemans, & de-Wit, 2017b)).

Thus far, all previous studies have used CFS to suppress two-dimensional (2-D) stimuli presented on a screen (an exception is one study that used virtual reality to present a CFS display and suppress virtual objects; van der Hoort, Reingardt, & Ehrsson, 2017). In a step toward a more ecological experience, Ludwig, Sterzer, Kathmann, Franz, and Hesselmann (2013) presented a setup in which haptic feedback was given for CF-suppressed virtual objects. Here we take an additional step, by introducing a novel variant of the classic CFS method that we term “real-life” CFS. The method allows suppression of real, three-dimensional (3-D) objects from awareness for periods of time comparable to those of classic CFS. Note that by using the term “real-life”, we are not implying that the method allows one to suppress the entire environment; as we explain below, currently it is limited to suppressing a portion of the visual field. Critically, however, this portion should suffice for experiments in which the subject can interact with the suppressed stimuli, which are real-life objects. In this article, we describe the details of this method and present two experiments in which it was used,^1^ as well as an additional experiment conducted using traditional CFS, in order to compare suppression between the novel variant we have developed and the traditional one.

## Suppressing real objects using “real-life” CFS—General description

Our method suppresses real objects from awareness by presenting Mondrians to the dominant eye using augmented reality (AR) glasses, while the nondominant eye is exposed to the real world. As in traditional CFS, when the Mondrians are presented to a region of the visual field in the dominant eye, the subject is temporarily incapable of consciously perceiving that region of the visual field in the contralateral eye, even though the region is presented there without any obstruction. Control of the graphics shown by the AR glasses is done by a computer, via a wireless connection. In order to display the graphics in the AR glasses with the same binocular disparity as the point in space at which the subject is looking, a short calibration procedure precedes the experimental session.

In the following paragraphs, we describe in general how the paradigm is implemented (for exemplary codes for the experiments, including the calibration and the experimental sessions, see the supplementary information) and how it should be used in future studies. Then we describe the specific methods used in each of the experiments included in this article, mainly focusing on the production and presentation of the relevant real-world stimuli.

### The setup

Our method is applicable to any type of glasses that can display graphics with varying levels of transparency. We used AR glasses produced by Epson (Moverio BT200) to present graphics that suppressed conscious visual perception of the real world. The resolution of the AR glasses was 960 × 540 pixels, with a refresh rate of 60 Hz and a color resolution of 24 bits, and an illumination of 700 cd/m^2^ when white pixels at 100% brightness were presented (manufacturer’s brightness specifications; an independent measurement we performed yielded even higher luminance values of ~ 850 cd/m^2^; see the supplementary information for more details). The graphics to be displayed with the glasses were produced by a computer using MATLAB (MathWorks, 2017) and the Psychtoolbox add-on (Brainard, 1997), though other software packages could also be used. Graphics were presented on a “virtual monitor” (“ghost monitor”), by extending the Windows desktop to an unconnected VGA output and using this monitor as Psychtoolbox’s target screen. This virtual screen was transmitted to the glasses online using a wireless LAN connection and the MirrorOp PC software and Android application (Awind, Inc.), which effectively mirrored what was displayed on the virtual screen to the AR glasses’ display. The AR glasses were set to “3D” mode, which split their display such that the left eye saw only the left half of the virtual screen, and the right eye saw the right half. In this way, the regions of the screen that were visible to each eye were easily distinguished as we programmed the experiment. Notably, since the display in the AR glasses mirrored the computer display via WiFi, there was a delay between the displays. To bring this delay to a minimum, we set the quality preferences using the MirrorOp software to be as low as possible. We estimate that this delay was of a magnitude of 400 ms, with some variability in timings (for details, see the supplementary information). Thus, we recommend that future studies take that into account when designing their experiment, as we have done (see the specific methods below).

### Stimuli

In our experiments, we used AR glasses to present the subject with a fusion frame (about 9.5 × 6.4 deg of visual angle; see the Calibration section below), a fixation cross, and the suppression stimuli (the “Mondrians”). The latter were circles ranging in radius from 0.21 to 0.84 deg of visual angle, in six different colors. All Mondrian colors were of 100% value and saturation on the HSV scale, differing only in their hue, to ensure that all Mondrians were presented with the highest possible opaqueness. Notably, since the virtual screen was presented to both eyes, half of the screen to each eye, the display was “stretched” in width. Therefore, the circle Mondrians appeared to the subject as somewhat elliptical, and vertical lines appeared somewhat thicker.

### Paradigm

#### Calibration

**Fig. 1:**
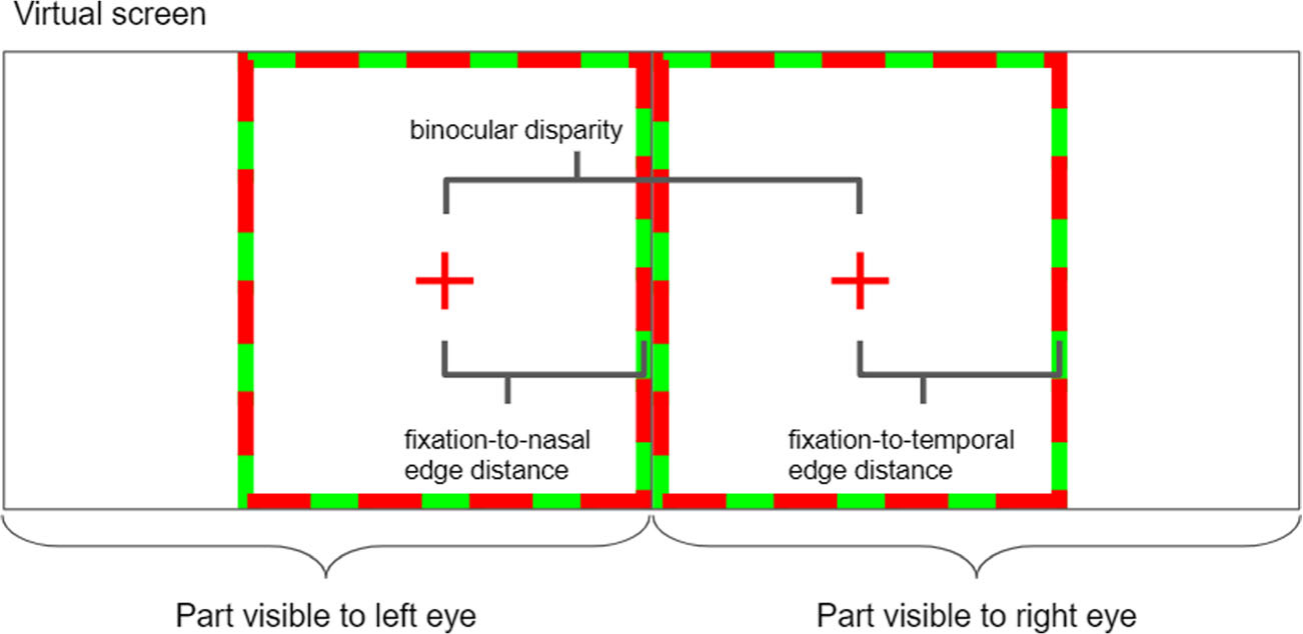
Illustration of the fusion frames following the calibration procedure, as they are presented on the virtual screen. When displayed in the goggles, this screen is divided so that each eye is presented with half of it. Red crosses serve as the fixation crosses, and the red and green frames facilitate fusion. Note that since the fixation is located exactly in the middle of the fusion frame, the fixation-to-nasal and fixation-to-temporal distances are equal.

After the subject put on the AR glasses, a calibration procedure was needed in order to calculate the correct binocular disparity with which the on-glasses graphics should to be presented. In “real-life” CFS, it is crucial that the subject’s binocular disparity match the location of the real-life stimuli that are about to be suppressed by the CFS stimulation. To reach such a match, a black fixation printed on a white placard was placed at the location of the to-be-presented real-life stimuli. In the glasses, one green fixation was presented to each eye. Using the mouse, the subject could control the distance between the two on-glasses fixations while keeping her gaze fixated on the real-world fixation. The subject was asked to change the distance between the on-glasses fixations and, if needed, to rotate her head, to reach convergence of all the fixation crosses—both the on-glasses ones and the real one. Once convergence had been achieved, the subject reported it by clicking the mouse button. The final position, in screen coordinates, of the on-glasses crosses was used as the center of the fusion frame. The width of the fusion frame was then set accordingly, setting the frame’s nasal edge along the middle of the virtual screen, and its temporal edge on the other side of the fixation cross and at the same distance as between the nasal edge and the fixation (Fig. 1).

***Trial*** Before trial onset, a real-world stimulus was positioned in front of the subject but hidden from the subject’s sight (see the description of our device used for real-world stimulus presentation in Exp. 1; different devices can, of course, be used according to the experimenter’s needs). At the beginning of each trial, a white background filled the fusion frames presented to both eyes for 2 s. In dim lighting conditions like those used in our experiments (see below, 2.75 cd/m^2^), the 700 cd/m^2^ illumination of the goggles means that they effectively turned opaque and no vision of the outside world was possible within the fusion frames (since the luminance measured inside our display box was two orders of magnitude less than the illumination provided by the goggles).

Mondrians were then flashed continuously at 10 Hz to the dominant eye, while the nondominant eye still saw the white background for 1 s. During this second, a window opened in our device, exposing the real-life stimulus. Notably, however, the subject was still not exposed to the stimulus, due to the effective opaqueness of the white background. Then the white background presented to the nondominant eye faded gradually to full transparency, over a period of 1 s. This fading mimicked the “ramping up” of the target stimulus’s contrast in classical on-screen CFS paradigms. Once the nondominant eye’s frame background was at full transparency, this eye was completely exposed to the real-world stimulus. Critically, however, due to the flashing Mondrians presented to the dominant eye, the stimulus was suppressed from awareness. Note that during Mondrian presentation, two factors render the difference in luminance between the content of the display box and the goggles smaller: The Mondrian display includes pixels with RGB values lower than those of pure white, and the real object in the box might reflect more light toward the subject’s eyes than the background. However, even the lowest luminance produced by the goggles while presenting the Mondrians was still two orders of magnitude greater than the brightness of the display box’s background (see the supplementary information, as well as a further discussion in the Discussion section below).

Depending on the research question and experimental requirements, the subject might then be asked to perform any sort of task relating to the suppressed stimulus: to report its properties in a direct manner and/or perform an indirect task that assessed its processing (Reingold & Merikle, 1988), to report when it was consciously perceived (e.g., for bCFS paradigms; Gayet et al., 2014), and even to interact with it. Posttrial subjective awareness might also be measured using a Perceptual Awareness Scale (PAS; Ramsøy & Overgaard, 2004) presented on the glasses (see the code in the supplementary information).

## Experiment 1: Validation of methodology and assessment of suppression effectiveness

**Fig. 2:**
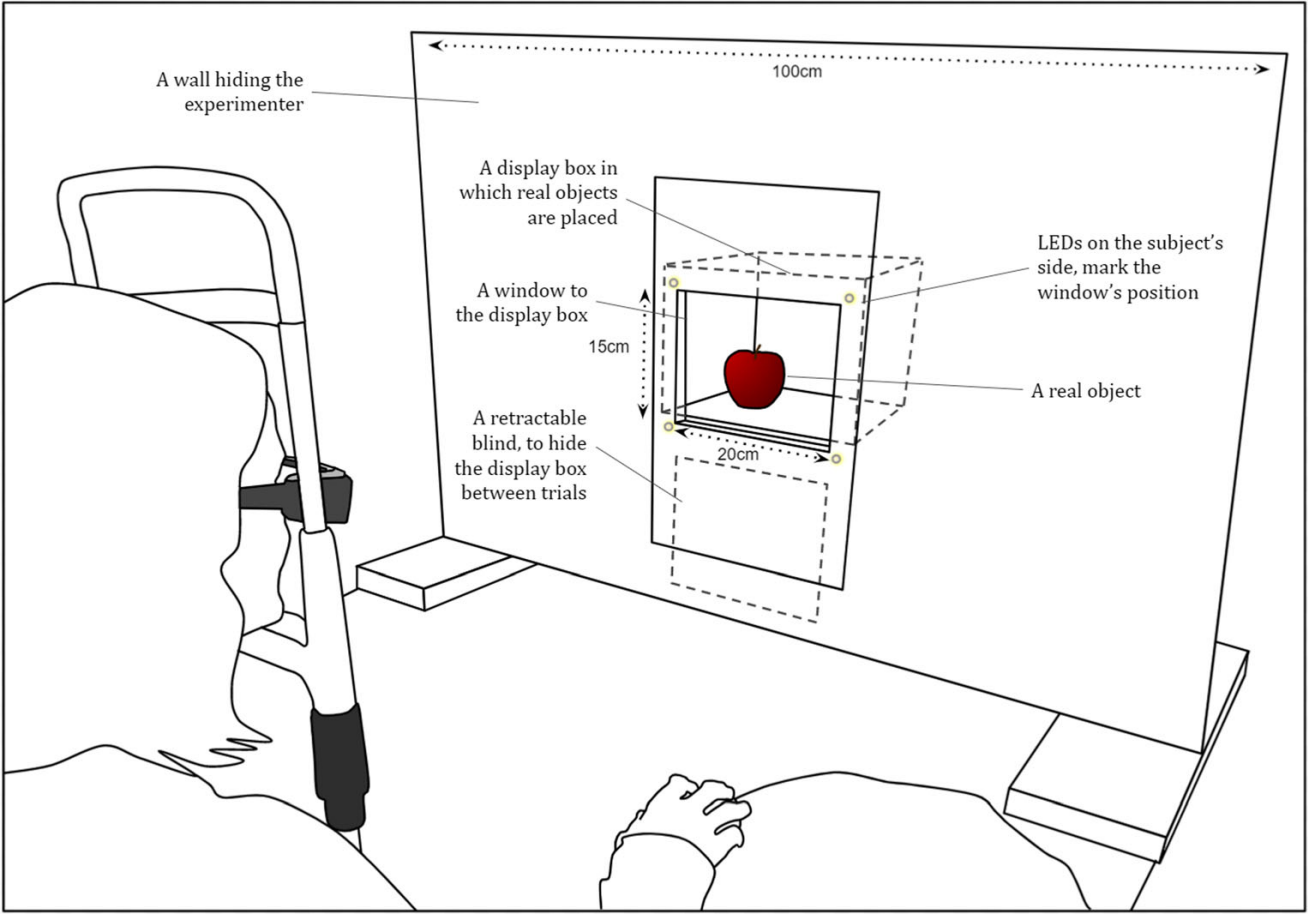
Schematic depiction of the experimental setup. The subject is sitting in front of the device, wearing the goggles, her head resting on a chin rest. The device is composed of a wall hiding the experimenter, a display box that includes a window, and a retractable blind. LEDs are used to mark the window position.

Because suppression of real objects using “real-life” CFS had never been done before, Experiment 1 was aimed at validating this methodology and making sure we could obtain effective suppression. We used “real-life” CFS to suppress both real objects and their 2-D image representations, which were printed color photos of the objects. A dedicated device (henceforth the “theater”; see Fig. 2) was constructed in order to display the physical (“real-world”) stimuli to the subject in synchronization with the on-glasses display. Subjects were first asked to report whether the physical stimulus (object or 2-D photo) was placed to the right or the left of the center of the theater display box (objective measure), and then to rate their level of awareness of the stimulus (subjective measure). We generally expected that the “real-life” CFS paradigm would be potent enough to evoke a large number of trials in which subjects reported not seeing the stimulus at all. We hypothesized that subjects’ performance would be at chance in those trials, regardless of the type of physical stimulus used (actual object/photograph). Notably, the hypotheses, analysis plan, sample size, and methods were all pre-registered at the Open Science Framework (OSF) website (https://osf.io/pgjf5/).^2^

### Method

#### Subjects

A total of 20 right-handed subjects participated in the experiment (10 females, 10 males; mean age = 25.79 years, *SD* = 3.75). The sample size was determined on the basis of the effect we found in Experiment 2 (see note 1; note, however, that the effect itself was less relevant to the present article, which is focused on the methodology and not the investigated effects), using the G*Power package (Faul, Erdfelder, Lang, & Buchner, 2007) with *α* = .05 (20 subjects translated to over 99% power).

Nine additional subjects who participated were excluded from the analysis: two due to technical difficulties (lights in the device not working properly or AR goggles malfunctioning); one due to not completing the experiment; four who reported less than 40 trials being “invisible” at each representation level (first exclusion criterion in the OSF pre-registration); one who reported the object location before the CFS display was removed (see the Procedure section below), while also reporting that he had not seen it, suggesting lack of understanding of the instructions or lack of willingness to follow them (third exclusion criterion in the OSF preregistration); and one due to pressing the same key throughout the experiment. Here and in all the following experiments, excluded subjects were removed before group-level analyses.

All subjects had normal or corrected-to-normal vision using contact lenses (optical glasses are uncomfortable to use with the AR glasses) and declared no past neurological, attentional, or mental disorders, or current use of psychiatric medicines. They participated in the experiment for credit or payment. Subjects signed a consent form and had explained to them that they could withdraw from the experiment at any point if they wished to do so. The experiment was approved by the university’s ethics committee.

#### Apparatus

The display theater device was devised so that the presented stimuli could be hidden from the subject between trials (Fig. 2). A computer was used to synchronize the opening of a blind installed in the theater device with the graphics presented by the glasses, such that the stimuli were exposed to the subject only while the CFS masks were displayed. A box containing a small stage was placed behind the blind, and the items were placed on the stage by an experimenter. The inside of the display box was lit by three sets of LED strips arranged along the inner side of the window and covered with parchment paper in order to scatter the light, so as to minimize highlights on the objects presented inside the box. The theater device was placed 90 cm away from the subject’s head, which was stabilized using a chin-rest.

As we explained above, the AR goggles were manufactured by Epson (Moverio BT200), and the experimental code was run using MATLAB (MathWorks, 2017) and the Psychtoolbox add-on (Brainard, 1997). Reporting was done using a mouse.

#### Real-world stimuli

**Fig. 3:**
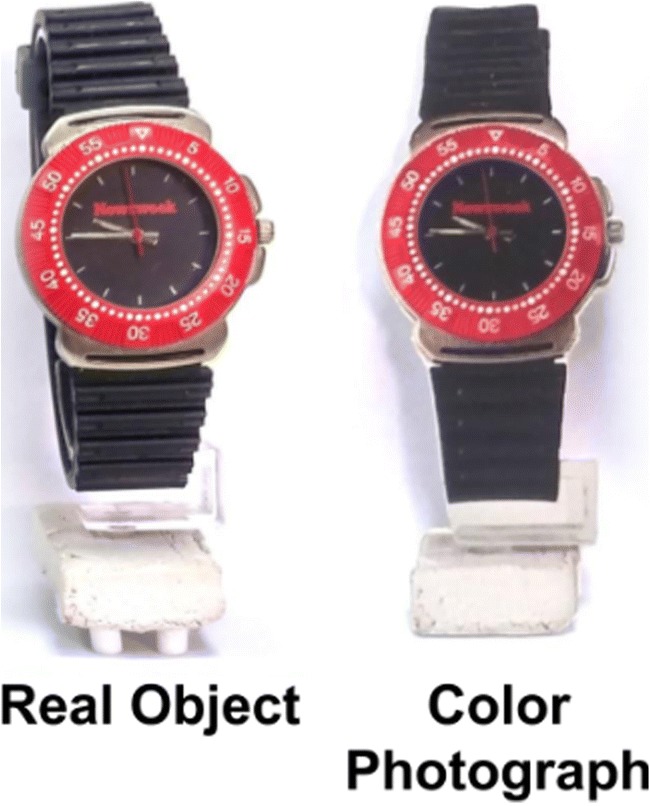
Examples of a real-world stimulus in both representation levels: the real object (left) and a cutout of a color photograph of the object (right). In Experiment 2, two more levels of representation were used, but they are presented and analyzed in another article.

The stimuli comprised 11 real-life, common, and easy-to-recognize objects, all less than 15 cm in width and 10 cm in height. Two-dimensional photographic reproductions (henceforth, “cutouts”) of the objects were created by photographing the objects inside the display box using a Canon 700D camera, from the subject’s point of view (Fig. 3). Each object was photographed when positioned both to the left and to the right of the box’s center, mimicking its possible positions in the experiment. The photographs’ colors and sizes were then digitally manipulated by a professional digital designer so that when printed, they would closely match those of the objects. Printing of the photographs was done on matte paper (Epson enhanced), to avoid further reflections. The positioning of the objects and cutouts inside the box was kept similar across trials by an array of pins attached to the box’s stage, upon which the objects or cutouts could be placed accurately in position and orientation.

#### Procedure

The experiment was composed of 176 trials divided into four blocks, with an additional training block (up to ten trials). Each experimental block thus consisted of 44 trials, in which all 11 objects were presented in all four experimental conditions: Representation Level (real object–color photo) × Side of the Box (left–right). The order of trials was set at the beginning of each block by a pseudo-randomization algorithm, with the constraint that the representation level or box side were not to be repeated on more than four consecutive trials. Between blocks, the subject could take a few minutes’ break. Each block started with a calibration of the on-glasses display (see the General Description above). Calibration was done with the inner LEDs of the theater device set to maximal intensity (yielding a background luminance of 58.5 cd/m^2^), such that the placard with the real-world fixation was seen at the best possible contrast.

##### Experimental blocks

To maximize CFS efficiency, the LEDs inside the theater device were dimmed to the minimal possible luminance (background luminance of 2.75 cd/m^2^), such that the contrast between the background and the stimuli was low. Before each trial, the experimenter closed the blind and placed a stimulus on one side of the box. The blind that hid the stage and the stimulus opened 400 ms after the command to start displaying the Mondrians was given by the computer, to account for the delay between the goggles and the computer (see above). A further precaution we took, to make sure the subject would never see the target stimulus prior to the Mondrian display due to this variable delay, was to present the 2-s white background stimulation. Accordingly, even if in some trials the blind fell before any Mondrians had been presented, the stimulus would not have been perceived because the white background effectively blocked its vision. During the trial, the on-glasses graphics were presented as described in the General Description section, with the CFS stimulation being presented for 4 s (including the first second, during which the opacity of the nondominant eye goggle was linearly decreased to 0%). The subject was asked to report the location of the stimulus as soon as possible, using the right and left mouse keys. If the subject detected the stimulus prior to the end of the 4-s period, CFS stimulation stopped. On the offset of the CFS masking (either upon the subject’s response or after 4 s), the experimenter quickly pulled the blind upward to hide the display box, and the on-glasses display’s background turned white (effectively opaque) in order to block any residual percept of the display box while it was being closed.

If the subject failed to report seeing the target stimulus until the CFS stimuli had ended, the background of the on-glasses display remained white for 4.6 additional seconds, prompting the subject to guess the stimulus position. Finally, the subject was presented with a subjective rating question using the PAS (Ramsøy & Overgaard, 2004). The question was presented on the glasses as four circles drawn on a white background, with a cursor controlled by the mouse, for subjects to use in selecting the most accurate description of their awareness of the stimulus, where 1 denoted *I saw nothing*, 2 represented *I saw a glimpse of something, but I couldn*’t *see what it was*, 3 stood for *I saw part of an object or a cutout*, and 4 signified *I clearly saw an object or a cutout*.

##### Training block

The goal of the training block was to introduce “real-life” CFS to the subject and allow her to experience the emergence of the stimuli into awareness. The trial sequence in the training block was identical to that in the experimental block, except for the following changes: (a) It had a longer CFS-only display (20 s), (b) the stimulus used was a single real object that was not used in the experimental blocks, and (c) the lighting of the theater was set to maximum intensity. After several trials, the experimenter dimmed the lights to the level that would be used in the experimental blocks. The experimenter ended the training block after at most ten trials, on the basis of the subject’s understanding of the paradigm.

### Results

**Fig. 4:**
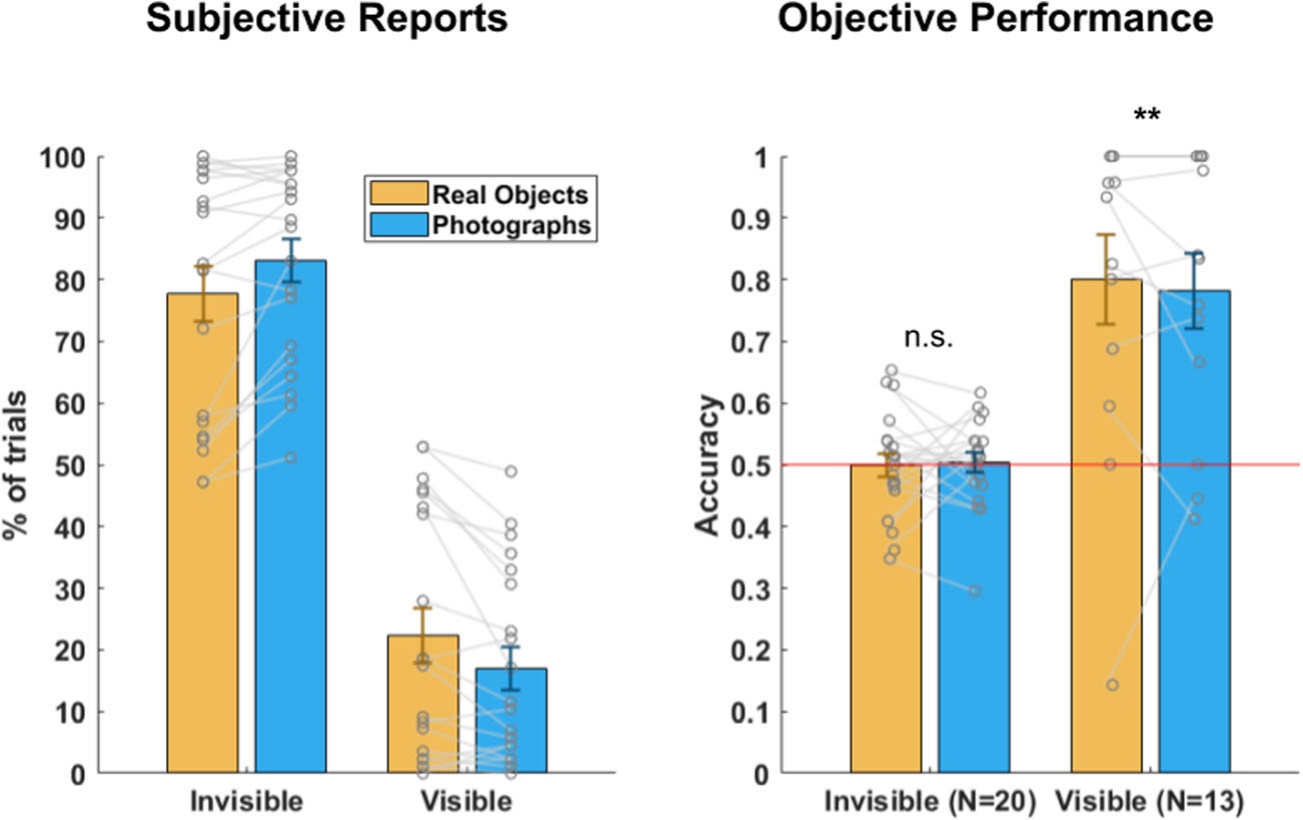
Means and standard errors of subjective reports of the visibility of the target stimuli (left), which were either real objects (blue) or cutouts of 2-D photographs (orange), and their corresponding objective performance (right). Note that trials with visibility 2–4 are grouped together as "visible". Data for individual subjects are denoted by dots. ^**^p < .0005: *t* test against chance.

The analysis was conducted using all trials in which subjects responded to the first question, about stimulus location, as requested (this was a forced choice task, and they were prompted to guess if they did not know the answer. Only a small fraction of the trials involved no response by the subject: 0.26%, on average, *SD* = 0.35). All analyses described here as confirmatory were predefined in the OSF presubmission, and all other analyses, conducted post-hoc, are marked as exploratory. The efficiency of “real-life” CFS was assessed by calculating the percentage of “visibility 1” trials in which subjects reported they did not see the stimulus. The mean percentage of such trials was 80.31% (*SD* = 17.15), demonstrating the potency of this suppression method (Fig. 4). Confirmatory analysis on the subjects’ performance at determining on which side the stimulus appeared showed that performance was at chance for photographs, as reflected by accuracy (*M* = 0.50, *SD* = 0.07; *t*(19) = 0.18, *p* = 0.86, Cohen’s *d* = 0.04 BF_10_ = 0.24; BF_10_ is the Bayes factor quantifying the evidence for the research hypothesis relative to the null hypothesis, as opposed to BF_01_, which denotes the evidence for the null hypothesis relative to the research hypothesis; see Fig. 4, right). As well, *d'* measures, estimating subjects’ sensitivity to the location of the stimulus (left vs. right) irrespective of the subjects’ criterion/bias, were calculated by treating the right side of the display as the “signal” and the left side as “noise,” and provided similar results [*M* = 0.07, *SD* = 0.30; *t*(19) = 0.99, *p* = 0.337, Cohen’s *d* = 0.22, BF_10_ = 0.357; hits and false alarm rates of 0 were replaced with 0.5/*n*, and those of 1 were replaced with (*n* – 0.5)/*n*, where *n* was the number of signal or noise trials, respectively (Stanislaw & Todorov, 1999)]. Performance was also at chance for real objects [accuracy: *M* = 0.5, *SD* = 0.084; *t*(19) = 0.017, *p* = 0.99, Cohen’s *d* = 0.004, BF_10_ = 0.23; mean *d'* = 0.05, *SD* = 0.48; *t*(19) = 0.49, *p* = 0.628, Cohen’s *d* = 0.11, BF_10_ = 0.26]. There was no difference in performance between photographs and real objects in those trials, in either accuracy [*t*(19) = 0.16, *p* = 0.875, Cohen’s *d* = 0.03, BF_10_ = 0.23] or *d'* [*t*(19) = 0.11, *p* = 0.912, Cohen’s *d* = 0.03, BF_10_ = 0.23]. In contrast, an exploratory analysis revealed that performance on trials with visibility scores of 2 or above (for subjects who had at least ten such trials) was well above chance (*M* = 0.798, *SD* = 0.219, *t*(12) = 4.717, *p* < 0.005 Bonferroni-corrected, Cohen’s *d* = 1.31, BF_10_ = 435,565; mean *d'* = 2.028, *SD* = 1.704, *t*(11) = 4.12, *p* = 0.014 Bonferroni-corrected, Cohen’s *d* = 1.19, BF_10_ = 25.65).

### Discussion

Experiment 1 demonstrated that the “real-life” CFS methodology is indeed successful at suppressing real-life 3-D objects (as well as cutouts of 2-D photographs of these objects). The vast majority of trials were rated by subjects as trials on which they did not see the target stimulus, and their ratings were further corroborated by their objective performance, which did not differ from chance. Critically, however, this experiment only showed that our method can suppress 3-D stimuli, without revealing the duration of such suppression. Accordingly, this was the goal of Experiment 2.

## Experiment 2: Assessment of suppression duration

In Experiment 2 we tested the span of suppression duration that can be achieved by “real-life” CFS,^3^ by presenting the Mondrians for a longer duration of 18.6s. A breaking-CFS paradigm was used (for a review, see Gayet et al., 2014), in which subjects were asked to report the side of the target stimulus as soon as they saw it. The subjects’ reaction times were therefore used as an estimation of the suppression duration achieved by “real-life” CFS.

### Method

#### Subjects

A total of 20 right-handed subjects participated in this experiment (14 females, six males; mean age = 24.35 years, *SD* = 3.88), all of whom met the predefined requirements for participation described above. Three additional subjects who participated were excluded from the analysis: two due to low accuracy (less than 75% correct), and one who did not complete the experiment due to technical difficulties. Because this study was exploratory (see note 1 above), the sample size was chosen on the basis of a common practice for exploratory experiments in our laboratory (*N* = 20). All subjects had normal or corrected-to-normal vision using contact lenses and declared no past neurological, attentional, or mental disorders, or current use of psychiatric medicines. They participated in the experiment for credit or payment. Subjects signed a consent form and had explained to them that they could withdraw from the experiment at any point if they wished to do so. The experiment was approved by the university’s ethics committee.

#### Stimuli, apparatus, and procedure

The stimuli, apparatus, and procedure were identical to those used in Experiment 1, aside from the following changes: First, the experiment included four levels of representation, ranging across (a) real, everyday objects; (b) cutouts of color photographs matched in color and size to the real objects; (c) cutouts of black-and-white photographs, created by reducing the saturation of the color photographs to zero; and (d) cutouts of contour-only photographs, created by employing a “find edges” filter on the black-and-white photographs (again, this was done as part of another research program, which we will publish elsewhere). For the purposes of this article and consistency with Experiment 1, we focus here only on levels (a) and (b). Accordingly, the experiment included 176 trials, divided into two experimental blocks, and an additional training block (of up to ten trials), all presented within one experimental session. Each experimental block thus consisted of 88 trials, showing 11 different objects in eight experimental conditions: Representation Levels (real object/color photograph/black-and-white photograph/contour-only photograph) × Sides of the Box (left–right).

Second, since this was a breaking-CFS paradigm, the flashing Mondrians appeared for 18.6s. The subject was asked to report seeing the stimulus by clicking the mouse button corresponding to the stimulus’s position relative to the center of the stage (left–right). After the subject had clicked a mouse button, or after 18.6s, the Mondrians disappeared and the subject could see the stage without interruption. Then the blind was lifted, and another trial began.

### Results

**Fig. 5:**
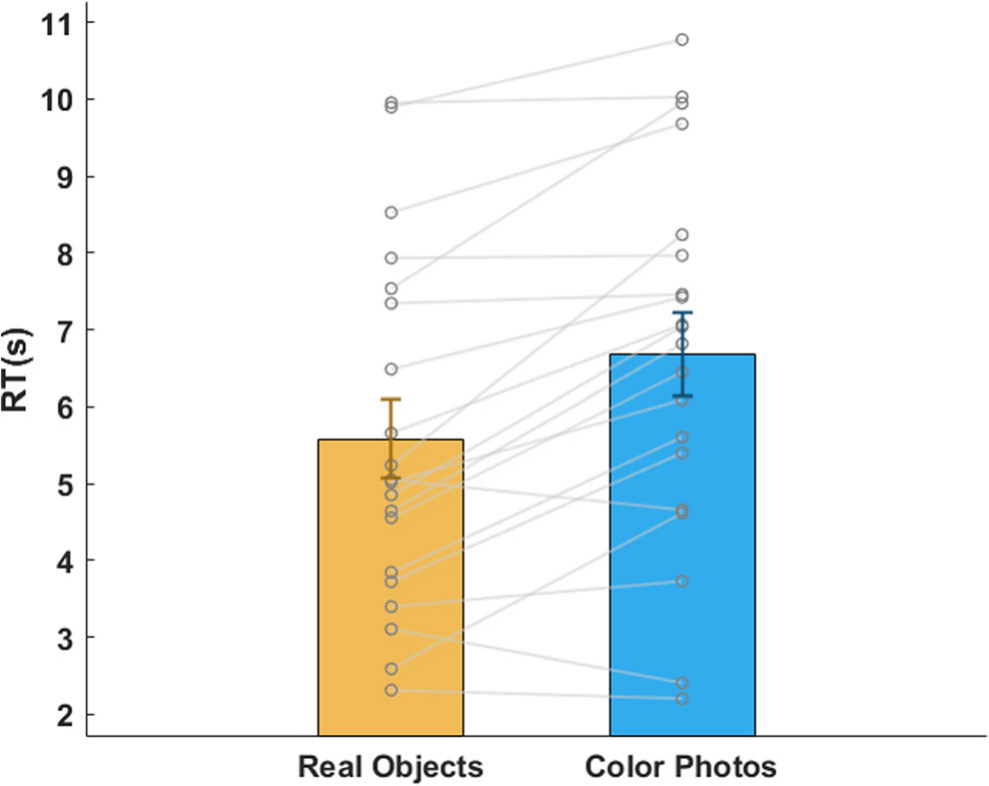
Suppression duration (means and *SE*s) for real objects (left) and cutouts of photographs (right). Data for individual subjects are denoted by dots.

In all of the following analyses, trials in which subjects gave the wrong answer about the stimulus location were excluded (6.8% of the trials). Accordingly, the included trials were either trials in which subjects were correct in their answer or trials in which no repose was provided, meaning that suppression was so strong that the stimulus did not break it. For the latter trials, the reaction time was considered as the whole length of the trial (18.6s). The average suppression duration was 6.38 s (*SD* = 2.42)—6.68 s for cutouts of photographs (*SD* = 2.42) and 5.58 s for real-life objects (*SD* = 2.29; see Fig. 5). To make sure the “no response” trials (constituting of 6.99% of the trials, *SD* = 5.42%) did not bias the distribution of reaction times (Kerr, Hesselmann, Räling, Wartenburger, & Sterzer, 2017), we conducted the same analysis on the data when excluding them, too, so that only trials on which subjects were correct in identifying the object location were included. The same pattern of results was found, though the suppression durations were obviously shorter (overall suppression: *M* = 5.26 s, *SD* = 1.97; photographs: *M* = 5.53 s, *SD* = 2.03; real objects: *M* = 5.00 s, *SD* = 1.96).

### Discussion

Once we had shown that “real-life” CFS can indeed suppress real-life objects, Experiment 2 provided a more fine-grained description of the suppression and its duration. We found that this suppression lasts, on average, 6.13 s (*SD* = 2.3), but it can also last for much longer; in our experiment, the display was removed after 18.6s, and on average, 6.99% of the trials ended without subjects being able to see the stimulus at all. In fact, in additional trials in the lab, as well as at public events (viz. the Vision Sciences Society demo night and another demonstration at the Association for the Scientific Study of Consciousness conference), the suppression sometimes lasted even for 30 s and was able to suppress not only a stationary stimulus, but also a moving human hand, or even a face. Though future studies will be needed to assess how potent this method is and to parametrically vary the factors that affect its potency, we can safely conclude that it is effective in suppressing stimuli, at least for several seconds. Yet, to directly compare its effectiveness with that of the classical on-screen method, we conducted Experiment 3.

## Experiment 3: Comparison of the suppression durations of “real-life” CFS versus classical CFS presented using a monitor

**Fig. 6:**
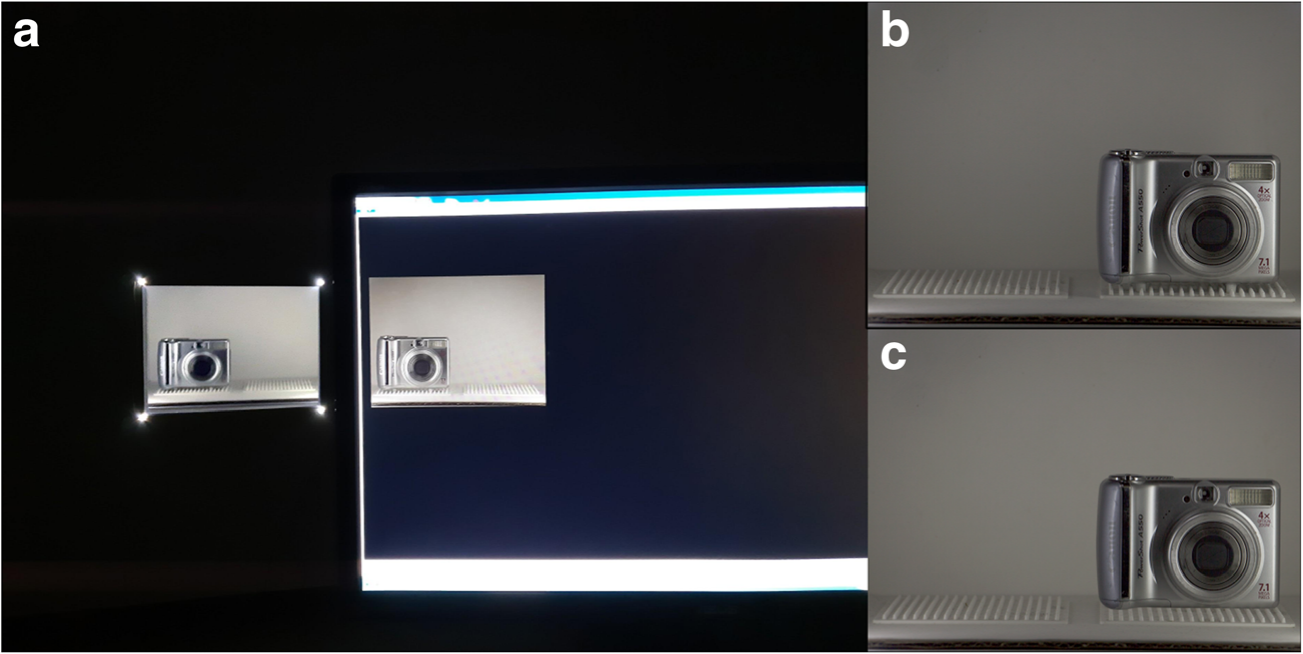
Preparation and examples of stimuli for Experiment 3. (A) The real display (left) versus the on-screen image (right). (B) Example of a stimulus from Experiment 3a—the object presented with its original background. That is, a photograph of the object placed in the theater display was presented as is. (C) Example of a stimulus from Experiment 3b—with the real background of the object replaced with a background that was similar in all the stimuli. That is, one photograph of the empty stage was used as the background, and the isolated objects were superimposed onto it.

In Experiment 3 we aimed to compare the novel “real-life” CFS with the well-known, widely used monitor-based CFS. We replicated the paradigm of Experiment 2 using very similar stimuli, which were now presented on a computer screen instead of as items in the real world. The real objects from Experiment 2 were photographed inside the display (Fig. 6a), and these images were then used as the target stimuli in a CFS paradigm presented using a monitor and a stereoscope (Fig. 6b). The suppression durations in this paradigm were compared to those obtained in Experiment 2.

Importantly, in classical CFS paradigms the target stimuli are usually presented on a uniform gray background. However, in “real-life” CFS this is not the case, since the objects have depth and therefore must be placed in a display box. Hence, in “real-life” CFS, nonuniform visual information other than the target stimulus is always present—for instance, the floor and back wall of the box. This environment is also influenced by the objects themselves, because they cast shadows on the walls and floor of the box. To make our monitor-presented experiment as similar as possible to Experiment 2, we tried to simulate the environment in which the stimuli were presented there. In Experiment 3a, we simply presented the photographs of the objects placed inside the box, so they appeared within the background in which they had appeared in Experiment 2, including their shadows and so on. In Experiment 3b, we wanted to control for individual differences evoked by each of the stimuli (e.g., the different shadows) and to more closely mimic classical CFS paradigms, in which the background is not at all influenced by the target stimulus. Thus, we used a photograph of the empty box and pasted the photographs of each isolated object onto that background photograph (see Fig. 6c).

### Method

#### Subjects

Twenty subjects participated in Experiment 3a (15 females, five male, 16 right-handed; mean age = 24.6 years, *SD* = 2.27). An additional subject was excluded because her performance level was at chance, indicating that she did not understand or follow the experiment instructions. Another subject was excluded because she did not provide any responses throughout the experiment. Twenty other subjects participated in Experiment 3b (13 females, seven males, 19 right-handed; mean age = 22.35 years, *SD* = 2.55). Here, too, one additional subject was excluded due to chance performance, and another due to no responses. The sample sizes were chosen to match that of Experiment 2.

All subjects had normal or corrected-to-normal vision and declared no past neurological, attentional, or mental disorders, or current use of psychiatric medicines. They participated in the experiment for credit or payment. Subjects signed a consent form and had it explained to them that they could withdraw from the experiment at any point if they wished to do so. The experiment was approved by the university’s ethics committee.

#### Stimuli

To prepare the stimuli for Experiments 3a and 3b, ten of the 11 original stimuli from Experiment 2 were photographed inside the theater device, on both sides of the box and with the lights at minimum intensity—identical to the one used in Experiment 2. These images then underwent a color-matching procedure using Photoshop (Adobe, Inc.), so as, when presented on a monitor in a dark room, to closely resemble the appearance of the real objects inside the theater device (Fig. 5A). In Experiment 3a, these images were used (Fig. 5B). For Experiment 3b, the background of each image, containing the floor and back wall of the box, was removed using Photoshop. A similar, identical background was inserted instead, which was the same for all images, such that the items appeared to cast no shadows on the display box (Fig. 5C). All images were scaled such that they would appear to be the same size as the real display, when presented at a distance of 60 cm from the subject.

#### Setup

The subject sat in front of a monitor placed at a distance of 60 cm from her eyes and with head rested on a chin-rest. A stereoscope was used to present each of the subject’s eyes with a different part of the screen. The subject performed the task using a mouse to report her experience and a keyboard to advance through the trials.

#### Paradigm

The procedure closely followed that of Experiment 2, except for the following changes: First, the maximum duration of suppression was 20 s. Second, instead of “ramping down” the opacity in the nondominant eye to expose it to the target stimulus, the nondominant eye was now presented with a white background that faded to the target image over the course of 1 s. Note that this mimicked the perceptual experience that subjects had in Experiment 2. Third, the total number of trials in the experimental block was 40, so that all ten objects were presented on both sides (right–left) for two repetitions. Since this experiment was very short (~ 20 min to complete), no break was given.

### Results

**Fig. 7:**
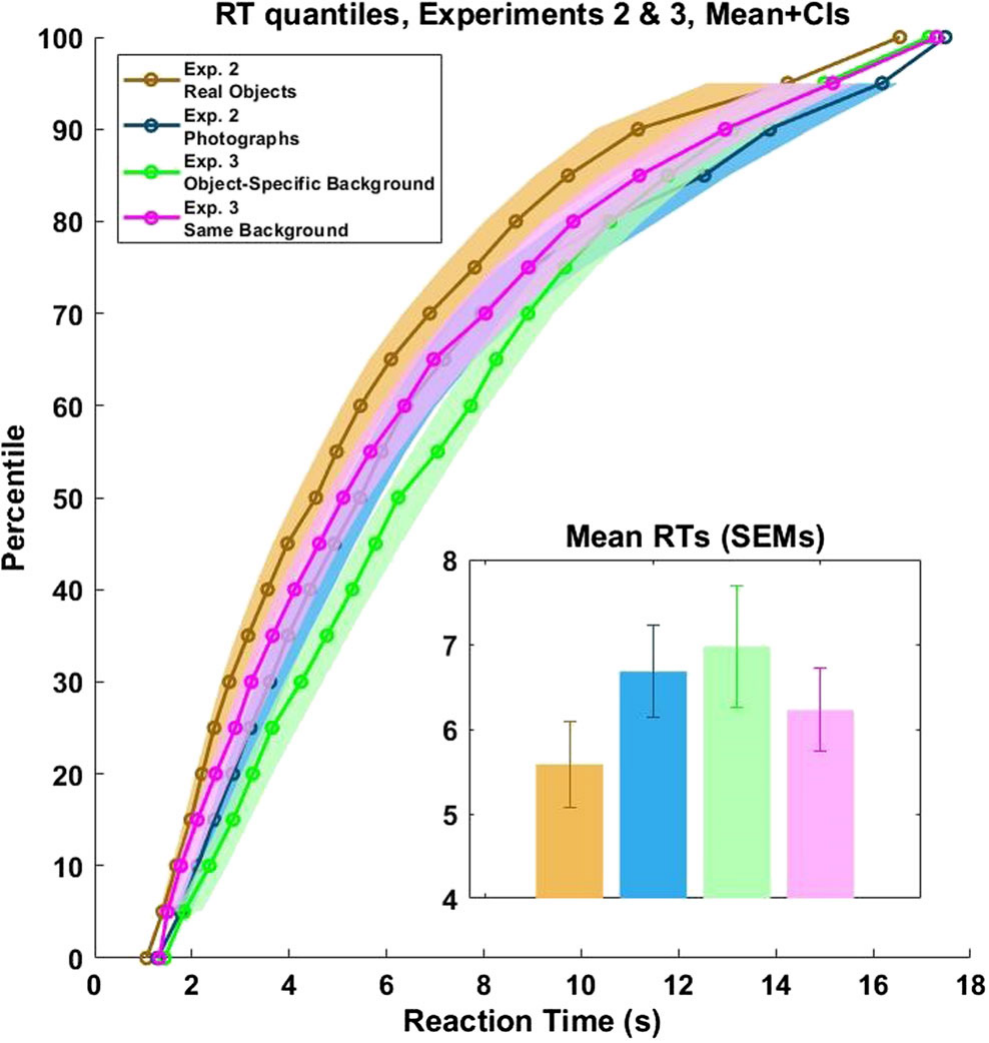
Vincentized display of the distributions of suppression durations in the “real-life” CFS Experiment 2 (objects, yellow; 2-D cutouts of photographs, blue) and in the on-screen versions (Exp. 3a, green; Exp. 3b, magenta). Polygons indicate 95% confidence intervals. The inset shows the mean and *SEM* of the suppression time in each condition.

The trial exclusion criteria were identical to those used in Experiment 2 (0.9% of the trials excluded due to wrong answers in Exp. 3a, 0.5% in Exp. 3b). The mean suppression time was 6.98 s in Experiment 3a (*SD* = 3.19; Fig. 7 inset), and 6.23 s in Experiment 3b (*SD* = 2.21; Fig. 7 inset). In 7% (*SD* = 11.9%) and 4.5% (*SD* = 6.2%) of the 40 trials (Exps. 3a and 3b, respectively), subjects did not see the stimulus at all. We here compare these times to those obtained in Experiment 2: 6.68 s (*SD* = 2.42) for photographs, and 5.58 s (*SD* = 2.29) for real-life objects. To better illustrate the distributions of subjects’ reaction times in both experiments, we present them in a vincentized display (Fig. 7; see also Stein, Hebart, & Sterzer, 2011, for a similar visualization). To that end, individual cumulative reaction time distributions were created for each subject. Then, the reaction times corresponding to the 1st through the 100th percentiles, at 5% intervals, were averaged across subjects to create group average reaction time distributions. We then generated 95% confidence intervals using bootstrapping. To compare the suppression times for the conditions in Experiment 2 to those in Experiments 3a and 3b, two analyses of variance (ANOVAs) were performed: The first compared the reaction times for real objects in Experiment 2 with the reaction times in both Experiments 3a and 3b; this allowed us to examine whether the suppression evoked by “real-life” CFS is different from classical on-screen CFS with respect to real, 3-D objects. The second analysis compared the reaction times for cutouts of photographs in Experiment 2 with the reaction times in both Experiments 3a and 3b. This focused on the comparison between “real-life” CFS and classical CFS, applied to 2-D stimuli—the photographs (notably, we could not compare all conditions in one ANOVA, because Exp. 2 had a within-subjects design, whereas Exps. 3a and 3b were conducted on separate and different samples). Both ANOVAs showed no statistical difference between “real-life” CFS and classical CFS [ANOVA #1: *F*(2, 57) = 1.44, *p* = .247, *η*^2^ = .048, BF_01_ = 2.586; ANOVA #2: *F*(2, 57) = 0.41, *p* = .67, *η*^2^ = .014, BF_01_ = 5.495]. Thus, “real-life” CFS is as potent as classical, on-screen CFS. We conducted the above analysis again after excluding the “no response” trials in both experiments, and found similar patterns [ANOVA #1: *F*(2, 57) = 2.283, *p* = .111, *η*^2^ = .074, BF_01_ = 1.396; ANOVA #2: *F*(2, 57) = 0.78, *p* = .47, *η*^2^ = .027, BF_01_ = 4.193].

### Discussion

Experiment 3 directly compared the potency of “real-life” CFS to that of the on-screen CFS paradigm. It did so by replicating Experiment 2 using an on-screen display, thus striving to achieve maximum similarity between the conditions and appearance of the stimuli in both experiments. This is of special importance, given the well-known effects of low-level features on suppression duration (Kaunitz, Fracasso, Skujevskis, & Melcher, 2014; Han, Blake, & Alais, 2018; Han, Lunghi, & Alais, 2016; Yang & Blake, 2012).

No difference in mean suppression durations was found when we compared both real objects and 2-D cutouts of photographs presented using “real-life” CFS to their representations presented using on-screen CFS. Moreover, this lack of evidence for a difference was found both when presenting the on-screen stimuli exactly as they had been seen in the real-life setup (Exp. 3a) and when presenting them in a manner closer to the characteristic figure–ground relations in on-screen CFS experiments (Exp. 3b). Of course, these null results, though strengthened by the Bayesian analysis we conducted, are not enough to determine that there is no difference between the two methods. However, such a difference—if it exists—seems to be minor. Thus, the “real-life” CFS suppression might be based on mechanisms similar to those elicited using classical, on-screen CFS. This implies that our method can indeed be used to study both unconscious processing and access to awareness of different types of stimuli (for a review, see, e.g., Gayet et al., 2014), thereby extending the range of possible research questions that can be investigated using CFS to the realm of 3-D, real stimuli.

## General discussion

In this article, we have presented “real-life” CFS—a novel variant of continuous flash suppression that allows, for the first time, suppression of real-world objects from awareness. This is different from all existing methods of unconscious presentation of stimuli, which only suppress 2-D on-screen representations of items, making it very hard, if not impossible, to conduct studies in which the subject can actually dynamically interact with the suppressed stimuli—which is uniquely enabled by our method. In three experiments, we showed that this new method is potent enough to allow prolonged suppression durations for stimuli that are otherwise easily visible. In Experiment 1, we provided a proof of concept and established the efficiency of the method in suppressing real stimuli. Subjects reported that they did not see the stimuli at all in about 80% of the trials. This subjective measure was further validated by their chance performance in determining the location of the suppressed stimuli (right/left) in these trials. Experiment 2 showed that this suppression was potent enough to last as long as 6.13 s across subjects. To compare this suppression duration with that in the classical CFS paradigm, in Experiment 3 we presented pictures of the same stimuli, now on-screen, and measured their suppression durations. The mean suppression times were indeed similar.

The above results thus provide compelling evidence for the ability of the “real-life” CFS method to suppress real-life stimuli from awareness. This is especially important, given the growing acknowledgment of the benefits of using more ecological stimuli in cognitive science in general. For example, in the field of face processing, studies have moved from schematic faces, to real-life images of them, and even to dynamic movies of faces (Bernstein, Erez, Blank, & Yovel, 2017; Pitcher, Dilks, Saxe, Triantafyllou, & Kanwisher, 2011); similarly, in language processing, more studies are focusing on real-life discourse, presenting long stories rather than isolated words or sentences (Silbert, Honey, Simony, Poeppel, & Hasson, 2014). Our methodology allows researchers to do the same in the field of consciousness studies, using real-life objects, and to design studies that will allow varying stimulus dynamics and, more importantly, to introduce questions about sensorimotor contingencies. Indeed, real-life, 3-D objects have been shown to elicit differential processing from 2-D representations of the same objects (Freud et al., 2016; Gomez, Skiba, & Snow, 2018; Snow et al., 2011; Snow, Skiba, Coleman, & Berryhill, 2014). This highlights the importance of expanding our investigation to real-life stimuli: First, given the differential processing that real-life objects might yield, different responses and new patterns of results might be found. It is accordingly timely to examine whether the results obtained with 2-D on-screen representations generalize to real-life objects. Second, because real-life objects might evoke stronger signals and effects (Gomez et al., 2018; Snow et al., 2011; Snow et al., 2014), using them as stimuli might increase our chances to find effects during unconscious perception. In other words, because on-screen 2-D stimuli are processed in a less direct and immediate manner than the actual entities that they represent, it is possible that real stimuli would be processed to a larger degree, and would accordingly evoke stronger effects. Given the inherent challenge of studying unconscious processing—overcoming the typically weak signal evoked by impoverished stimuli (Greenwald, Draine, & Abrams, 1996)—this might be a fruitful venue of research.

The novel methodology we report here has its limitations, of course, which mainly stem from the current technological constraints of the AR hardware used for displaying the Mondrians. For example, at present the technique cannot mask the entire real-world environment around us, but only a somewhat limited area within the visual field, namely the area that can be presented with graphics. This becomes an issue when trying to suppress objects that are very close to the subject or that are very big. In addition, because eye-tracking solutions for AR are not yet common, the subject’s head and its distance from the suppressed object have to be fixed. Another potential issue is that of leakage—that is, residual perception of the suppressed stimulus by the dominant eye, which is supposed to see only the Mondrians. Potentially, light reflected off the to-be-suppressed objects may be strong enough to be seen by the eye to which the Mondrians are presented. Such crosstalk between the eyes (see Baker, Kaestner, & Gouws, 2016; see also Hesselmann, Darcy, Rothkirch, & Sterzer, 2018; Rothkirch & Hesselmann, 2018) can be avoided by simply placing a cover in front of the glasses on the side of the dominant eye, over the region of the visual field where the Mondrians are to be presented. Such a cover would prevent any information from the outside world penetrating the Mondrian display and accordingly reaching the dominant eye. Finally, for the specific goggles used here, there was a certain delay between the goggles and the computer, causing a delay in presentation times, as well as some jitter (see the supplementary information for further details), which renders our technique less useful for paradigms that require short stimulus presentation times. However, because advances in AR technology are common and rapid, we hope it will be possible to overcome these drawbacks soon.

Other limitations pertain more to the real-life objects that can be used as stimuli. First, for practical reasons, the set of stimuli used in such experiments will tend to be smaller than those used in experiments with computer-generated stimuli. It may thus be fitting to use mixed-effects model analysis with stimulus as a random factor, to assure that the effect of the variables in question is not being driven by a specific subset of the stimuli used. Second, this setup makes it harder to implement one of the widely used control conditions in breaking-CFS studies, in which the Mondrians are presented to both eyes, with the target stimulus overlaid on them. This control condition aims at differentiating between the effects of CFS-specific processing and effects stemming from different detection thresholds or other postperceptual processes in the different conditions. A possible solution could be to present the Mondrians to both eyes and to gradually reduce their contrast. Note, however, that this would not isolate the critical object, but rather measure the time it takes the entire environment (within the designated visual field) to break suppression. In addition, this control condition has already been substantially criticized, due to both the differences in the reaction time distribution between this condition and the experimental one and the difference in subjective experience between the conditions (Stein et al., 2011). Therefore, other (or modified) control conditions have been suggested that might be more suitable (Gayet et al., 2014). These additional controls (i.e., disrupting stimulus meaning or focusing on stimulus reportability) can also be conducted with the present method.

Even with its current limitations, “real-life” CFS opens the way for many new possibilities in the study of unconscious processing. Essentially, anything in the real world can in principle be suppressed (notwithstanding the above-mentioned reservations). Already now it is possible, for example, to suppress animate, moving stimuli, such as hands and faces. Likewise, real, manipulable objects can be suppressed from awareness, permitting the study of unconscious processing of affordances, and even designs that involve actual interactions between the subject and the suppressed stimuli. The difference in unconscious processing between 3-D objects and 2-D representations can also be studied, as we have done in the reported experiments (we will discuss these results elsewhere, and thus focus here on methodological aspects). Importantly, and unlike in virtual reality solutions (van der Hoort et al., 2017), the ecological validity of our method is much greater. It is the first suppression method that allows fully immersive, real interaction with the subject’s surrounding environment, without having to re-represent it in any way. Accordingly, our technique gets us one step closer to the common stimuli the brain has to process in real, everyday life.

### Author note

This study was supported by the Israel Science Foundation (Grant No. 1847/16). The authors thank Alexander Kolominsky for his help in creating the stimuli used in the experiments.

## Electronic supplementary material

ESM 1 (DOCX 20.9 kb)

ESM 2 (DOCX 13.6 kb)

ESM 3 (DOCX 60.8 kb)

ESM 4 (DOCX 216 kb)

## Footnotes

1 Though originally we conducted Experiment 2 prior to Experiment 1 (since Exp. 2 was part of a larger-scale study that we will report elsewhere), we present them here in reversed order so as to facilitate their understanding.

2 The OSF pre-registration includes hypotheses about the difference in visibility between the 2-D and 3-D stimuli. Because this article focuses on the methodological aspects of our work, we do not refer to this hypothesis here. A description of that research question, and of additional studies conducted to address it, will be published elsewhere.

3 As in Experiment 1, this experiment also examined the difference in suppression efficiency between real objects and photographs. The latter theoretical question was a part of larger-scale research that will be discussed elsewhere, since it exceeds the scope of the present article. We therefore focus here only on the methodological aspects of this experiment.
