# Supplementary material for: “Real-life” continuous flash suppression (CFS)-CFS with real-world objects using augmented reality goggles
Source: Behav Res Methods. 2018 Nov 14;51(6):2827–39. doi: 10.3758/s13428-018-1162-0 (PMC6877487; doi:10.3758/s13428-018-1162-0)
Supplement: Supplementary file 1 — (DOCX 20.9 kb) [file 13428_2018_1162_MOESM1_ESM.docx]

# Supplementary Information

This document lists the files added as supplementary information for this paper.

## Raw data

We provide the raw data for experiments 1, 2 and 3. The structure of each data file is portrayed below.

### Experiment 1

File downloadable from: <https://osf.io/kg8b2/download>

Sheet: “Raw_Data_ResponseTrials”. The data presented is of all the trials in which subjects gave a response. Each row is a single trial from a single subject. Columns:

| Column Name | Content |
| --- | --- |
| Subject_ID | Subject’s identifying number |
| Session | Session number. The experiment was done in one meeting, divided into four sessions, with short breaks between them. |
| Block | Block number within session. For most sessions, there is only one block. For sessions that were stopped abruptly and continued later, there are two blocks. |
| Trial | Trial number within block. |
| Code | Trial code, representing the conditions in this trial. First digit is stimulus position (1=Left, 2=Right), second digit is representation level (1=Real object, 2=Photograph). See two following columns. |
| Item_Position | Where, in this trial, the stimulus is presented on the stage. “Subject Right” means that it is on the right side, from the subject’s point of view. “Subject Left” means it’s on the left. |
| Representation_Level | The representation level of the stimulus in this trial. Could be either a real object or a color photograph of this object. |
| Stimulus_ID | A number identifying the stimulus in the current trial |
| Stimulus_Name | The name of the stimulus in the current trial |
| Fixation_Onset_timestamp | Time of fixation onset, in seconds. The reference zero point is arbitrary – this is only for comparison between trials or events inside a trial. |
| Shutter_Drop_timestamp | When the blind fell down to reveal the stage, in seconds. The reference zero point is arbitrary – this is only for comparison between trials or events inside a trial. |
| RT | Subject’s reaction time in this trial. Measured from the beginning of the trial. |
| Subject_Resp_Key | The mouse button on which the subject pressed. 1=Left, 2=Right. |
| Subject_Resp_Side | Stimulus position as reported by the subject. A translation of the previous field to either “Left” or “Right”. |
| PAS_startTime | Time of Perceptual Awareness Scale appearance, in seconds. The reference zero point is arbitrary – this is only for comparison between trials or events inside a trial. |
| PAS_choice | Subject’s choice in the Perceptual Awareness Scale. 1 denotes “I saw nothing”, 2 represents “I saw a glimpse of something, but I couldn’t see what it was”, 3 stands for “I saw part of an object or a cutout”, and 4 signifies “I clearly saw an object or a cutout” |
| PAS_RT | Subject’s reaction time to the Perceptual Awareness Scale, in seconds. |
| Correct | Whether the subject’s response in this trial was correct. |

### Experiment 2

File downloadable from: <https://osf.io/mgyj7/download>

Sheet: “Raw_Data_CorrectTrials”. The data presented is of all the trials in which subjects either gave a correct response, or no response at all. Each row is a single trial from a single subject. Columns are the same as in Experiment 1, except for:

| Column Name | Content |
| --- | --- |
| Session | Session number. The experiment was done in one meeting, divided into two sessions, with a short break between them. |
| Code | Trial code, representing the conditions in this trial. First digit is stimulus position (1=Left, 2=Right), second digit is representation level (1=Real object, 2=Photograph, 3=Black & white photograph, 4=Contour image). |
| Representation_Level | The representation level of the stimulus in this trial. Could be a real object, a color photograph of this object, a black-and-white version of this photograph, or a contour-only version of this photograph. |
| RT | Subject’s reaction time in this trial. Measured from the beginning of the “ramping down” in opacity of the white background presented to the non-dominant eye. |
| Subject_Resp_Key | The mouse button on which the subject pressed. 1=Left, 2=Right, empty = No response. |
| Subject_Resp_Side | Stimulus position as reported by the subject. A translation of the previous field to either “Left”, “Right”, or “[]”, which represents no response. |
| Response? | A binary marking whether there was a response in this trial. 0=No response, 1=Response. |

Notice: No PAS was presented in this version of the experiment.

### Experiment 3

File downloadable from: <https://osf.io/f4na8/download>

Sheets: “RawData_Exp3a_CorrectTrials”,” RawData_Exp3b_CorrectTrials”. The data presented is of all the trials in which subjects either gave a correct response, or no response at all. Each row is a single trial from a single subject. Columns are the same as in Experiment 1, except for:

| Column Name | Content |
| --- | --- |
| Session | Session number. The experiment was done in one meeting, and there was only one session. |
| Code | Trial code, representing the stimulus position (1=Left, 2=Right). |
| RT_without_cutoff | Subject’s reaction time in this trial. Measured from the beginning of the “ramping down” in opacity of the white background presented to the non-dominant eye. |
| RT_with_cutoff | Subject’s reaction time, cutoff to 18.6s, to match maximal CFS time in Experiment 2. |
| Subject_Resp_Key | The mouse button on which the subject pressed. 1=Left, 2=Right, empty = No response. |
| Subject_Resp_Side | Stimulus position as reported by the subject. A translation of the previous field to either “Left”, “Right”, or “[]”, which represents no response. |
| Response? | A binary marking whether there was a response in this trial. 0=No response, 1=Response. |

Sheet “Comparison_Exp2_Exps3a,b”. Each row is the mean reaction time of a single subject in a specific condition (for Experiment 2), or simply of a single subject (for Experiment 3a,3b). Columns:

| Column Name | Content |
| --- | --- |
| Subject_ID | Subject’s identifying number in the relevant experiment. |
| RT_mean | Subject’s mean reaction time in the relevant experiment and condition. |
| Experiment | The experiment and condition which this line describes. |
| #Trials | The number of trials upon which “RT_mean” was calculated |
| Cond_enum | An enumeration of the “Experiment” column, for easier access. |

## Estimation of the computer-to-AR goggles timing differences

“SI_GogglesConnectivity” - A document characterizing the delay between the computer and the augmented-reality goggles, with estimation of its variability.

## Measurement of the AR goggles’ luminance output

“SI_GogglesBrightness” – A document characterizing the intensity of luminance provided by the AR goggles, for the different RGB values used in our experiments.

## Experiment code and manual

Zip file of the experiment code downloadable from: <https://osf.io/vkgjf/download>

We provide the code for running our experiment, and adapt it to your needs. The file “Real_Life_CFS_code.zip” contains the folder with the experiment code. To install it and run the experiment, read the attached manual: “SI_codePackageManual”.
