# Supplementary material for: “Real-life” continuous flash suppression (CFS)-CFS with real-world objects using augmented reality goggles
Source: Behav Res Methods. 2018 Nov 14;51(6):2827–39. doi: 10.3758/s13428-018-1162-0 (PMC6877487; doi:10.3758/s13428-018-1162-0)
Supplement: Supplementary file 2 — (DOCX 13.6 kb) [file 13428_2018_1162_MOESM2_ESM.docx]

### Estimation of the computer-to-AR goggles timing differences

To better assess the computer-goggles delay and its variability, we used an oscilloscope. A photodiode that was attached to the AR goggles’ display and an LPT-to-BNC wire that transmitted triggers directly from the controlling computer were both connected to the oscilloscope. We displayed trains of 40 full-screen flashes on the virtual screen by alternating the color of the whole screen between black and white, at a rate of 192ms per flash (half cycle white), using MATLAB and Psychtoolbox. Ten such trains were displayed and measured. Each command to alternate the screen color was accompanied by a trigger sent from the computer to the oscilloscope, and a respective spike in the input received from the photodiode. Using an in-house written MATLAB script, we measured the difference in time between each such pair of events, which represents the delay between the computer and the AR goggles’ display. Importantly, as Psychtoolbox occasionally “misses” frame alternations (fails to carry out a command to change the virtual monitor’s display in the specific time it was supposed to), some of the trains recorded by the photodiode had one or two flashes missing from the beginning of the train - representing a failure on the computer’s side to present the graphics, and not a failure of the WiFi connection to transmit them. The delay measured without taking these misses into account had a mean of **346**ms (SD=**126**), calculated by pooling the delays from all the trains. The delay representing only the difference in timing between the virtual screen and the AR goggles had a much lower mean of **138**ms (SD=19; Figure S1).


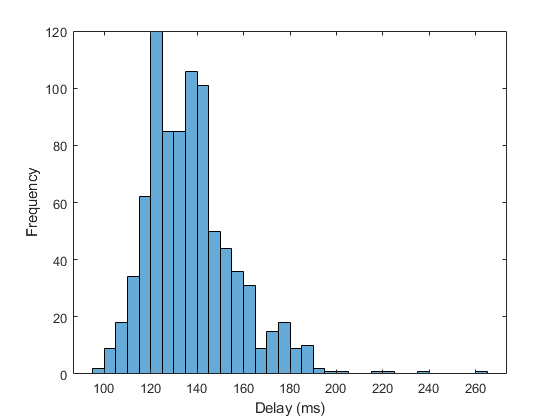


Figure S1 - Distribution of delay times between the virtual screen and the on-goggles display, excluding flashes that were sent by MATLAB but were missed on the virtual screen. Total N=852.
