# Supplementary material for: “Real-life” continuous flash suppression (CFS)-CFS with real-world objects using augmented reality goggles
Source: Behav Res Methods. 2018 Nov 14;51(6):2827–39. doi: 10.3758/s13428-018-1162-0 (PMC6877487; doi:10.3758/s13428-018-1162-0)
Supplement: Supplementary file 3 — (DOCX 60.8 kb) [file 13428_2018_1162_MOESM3_ESM.docx]

Measurement of the AR goggles’ luminance output

In our experiments, we use the AR goggles to present the subject’s dominant eye with a white background at the beginning of the trial, and flashing Mondrians afterwards. The subject’s non-dominant eye is presented with a white background which gradually becomes transparent, and is thus exposed to the target stimulus (a real object). While the dominant eye is not supposed to be exposed to the target stimulus at all, a stimulus which is bright enough could potentially shine through the white\Mondrian display presented to the dominant eye. This could cause some level of crosstalk between the eyes, in which the dominant eye is at least partially exposed to the target stimulus. To assess the light intensity differences between the on-glasses graphics presented to the dominant eye and the light reflected off the target stimulus, we measured the luminance produced by the goggles for the different colors used for the Mondrians and for pure white, and compared it to the light intensity inside our theater device when empty.


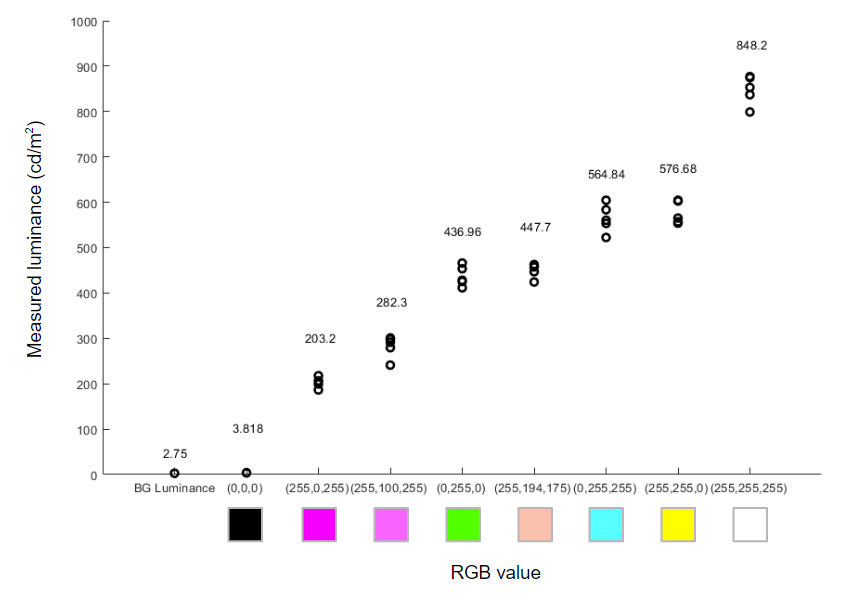


Figure S2 - Luminance produced by the BT-200 MOVERIO AR goggles for the different RGB values used in our experiments. Luminance was measured using a photometer in a dark room from a distance of 1m, with the goggles at 100% brightness setting. Each RGB value was measured five times, by setting the whole display of the goggles to this value and pointing the photometer to one of the goggles’ lenses. For each RGB value, the five measures are plotted, and their mean is noted above them. The leftmost column shows the brightness of the display box’s background (“BG Luminance”) under the experiment’s lighting conditions. The second column from the left shows the luminance output of the goggles for pure black, which is not a color used when presenting the Mondrians, but rather the RGB value for which the goggles become the most transparent (presented here for completeness).
