# Supplementary material for: “Real-life” continuous flash suppression (CFS)-CFS with real-world objects using augmented reality goggles
Source: Behav Res Methods. 2018 Nov 14;51(6):2827–39. doi: 10.3758/s13428-018-1162-0 (PMC6877487; doi:10.3758/s13428-018-1162-0)
Supplement: Supplementary file 4 — (DOCX 216 kb) [file 13428_2018_1162_MOESM4_ESM.docx]

**Real-Life CFS MATLAB Script - Basic Instructions**

This document describes how to install, prepare and run the MATLAB script of the Real-Life CFS paradigm. It also provides some instructions about how to alter the experiments to suit your needs. If you encounter problems, you may contact: [urikorisky@tauex.tau.ac.il](mailto:urikorisky@tauex.tau.ac.il)

**TOC**

[Requirements](#_h1kjph5pjlgd)

[Installation](#_qmt5091vzq)

[Preparing the display](#_22p9j72vjdmg)

[Windows 7 - Extending to a “ghost” monitor](#_muopnjhku1vo)

[Windows 10 - Extending to a “ghost” monitor](#_hnyic7d0x2hp)

[Running the experiment](#_4g8hn71q0dbc)

[Troubleshooting](#_pl6i4l8f4msz)

[The parameters file](#_da5ijr4vcstz)

[The “Params” sheet](#_l4bd80jn5gor)

[Properties of the CFS sequence](#_uhpb7vhm5mei)

[Properties controlling experiment flow](#_if3dx14eaa45)

[Basic introduction to the code](#_y41nq0hrcjnj)

## Requirements

- MATLAB v. 2016a and above
- The Psychtoolbox add-on for MATLAB, downloadable from:
  <http://psychtoolbox.org/>
- Mex-File Plug-in for Fast MATLAB Port I/O - for controlling devices through LPT ports. Follow installation instructions on:
  <http://apps.usd.edu/coglab/psyc770/IO64.html>
- A software or a connection allowing mirroring of the computer screen to the AR goggles. For example, MirrorOp:
  <http://www.mirrorop.com/>

**This script was tested on Windows 7 with MATLAB 2017b**. It should work on older versions of MATLAB, and on newer versions of Windows, but it is not guaranteed. The procedure of screen extension may differ on Windows 10 (see below).

## Installation

Unzip the package to a folder of your choice. Direct MATLAB’s current working directory to that folder. You don’t need to add this folder to MATLAB’s path.

## Preparing the display

To present the graphics produced by the script on the AR goggles, but still be able to see MATLAB’s command window, you need to extend your display such that you will have two screens: the main one, showing MATLAB, which is presented on a real monitor, and a second, which can be a “virtual” or “ghost” one, and will be used by Psychtoolbox (PTB) to draw the graphics. This second screen will be mirrored to the AR goggles.

See instructions below on how to run the experiment without this procedure. Note that this means you will not be able to see the MATLAB screen, which includes necessary information for the experimenter, while the experiment runs.

### Windows 7 - Extending to a “ghost” monitor

1. Right-click on the desktop and choose “Screen resolution”
2. If you have only one monitor connected, you will see an illustration of one monitor:

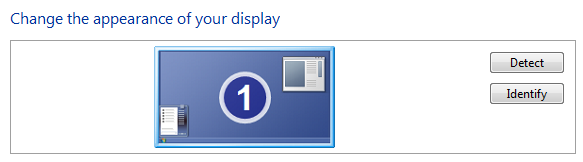

3. Click “Detect”. You will see an option for connecting another screen (or more than one):

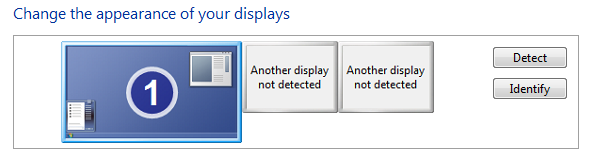

4. Click one of the gray rectangles that have “Another display not detected” written on them. In the “Multiple displays” option below, choose: “Try to connect anyway on: VGA”

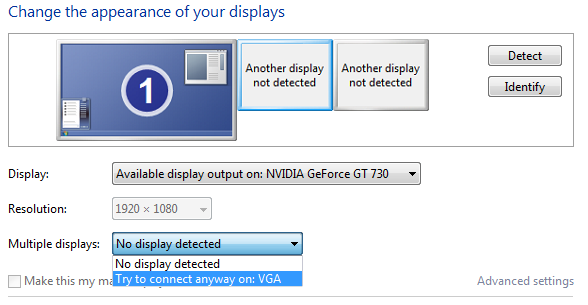

5. This display will now have “2” written on it. Click “Apply”.
6. Click the second display and under “Multiple displays” select “Extend these displays”. Click “Apply” and then “Keep Changes”.

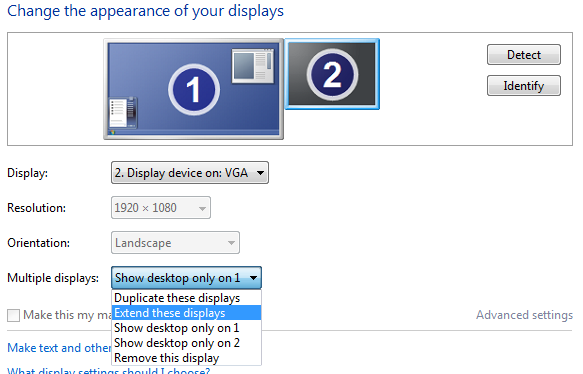

7. The “ghost” monitor is now set up and ready to use. You can mirror it to your AR goggles and check that you see it clearly.

### Windows 10 - Extending to a “ghost” monitor

On Windows 10, it may be impossible to connect a virtual monitor. One possible solution is to connect an actual second monitor and extend the display to it. Another possibility which may work if you have an HDMI port, is to connect the main monitor to the VGA\DVI port, and connect an HDMI-to-VGA converter to the HDMI port, without connecting anything to the converter. This “tricks” Windows 10 into thinking a second monitor is actually connected, allowing the extension of the display onto this “fake” display too.

## Running the experiment

After you prepared the displays and mirrored the “ghost” display to the AR goggles, open MATLAB and follow these instructions:

1. Change the current working folder to the folder in which the script was installed. You should see only one script file in this folder: “RunExp_RealLife_CFS.m”. Run this script.
2. A dialog box will open, asking you to choose a parameters file for this session. The default location for parameter files is the subfolder “Stim”. Choose the parameter file you wish to run. The example code comes with the following possible experimental settings, which adhere to the experiments described in the main text:
   1. Experiment 1: On each trial, CFS masks are displayed for a total of 5 seconds, and the subject’s non-dominant eye is exposed to the real world for the last 4 seconds. The subject has to click the mouse button to indicate where the suppressed stimulus was placed. If the subject doesn’t answer until the suppression time is over, the background becomes opaque (white) again and they are given 5 seconds to guess. A perceptual awareness scale (PAS) is displayed after the subject answers.
   2. Experiment 2: CFS masks are displayed for a total of 20 seconds, and the subject’s non-dominant eye is exposed to the real world for the last 19 seconds. The subject has to click the mouse button to indicate where the suppressed stimulus was placed. Once the subject answers, or the suppression time is over, both of the subject’s eyes are exposed to the real world.
   3. Experiment 3: An on-screen version of the experiment. The CFS masks appear for 20 seconds. After the first second, there is a “ramping up” of the image to the non-dominant eye. Note that this version needs to run with only one, real display.
3. The script will ask you to provide the subject ID in the command window. If you input “0”, the experiment will enter a test mode. This mostly means that no calibration will be performed.
4. The script will ask you to provide subject age, dominant eye, dominant hand and sex. Of all these properties, only “dominant eye” affects the rest of the experiment, and the others serve to document subjects’ characteristics. Input “r” or “l”.
5. Wait until you see the writing “Fusion test began” in the command window, notifying you that the calibration phase has started. Perform the calibration:
   1. Put a placard with a cross painted on it in the same distance from the subject as your stimuli will be.
   2. The subject will now be presented with one green cross to each eye. By moving the mouse forwards and backwards, the subject pushes the green crosses together or pulls them apart, respectively.
   3. Ask the subject to focus on the real cross, on the placard, and to move the green crosses (by moving the mouse) until both green crosses merge with the real cross. Once the subject manages to do that, they should click the mouse button.
   4. Once the subject clicked the mouse button, the message “Fusion test ended” will appear. The subject will see two red frames with red “X”’s, until the script finishes preparing the graphics for presentation.
6. Wait until the writing “Trial ready. Press ENTER to start.” appears in the command window. You may now press ENTER whenever the target stimulus is in place, to start the trial.
7. After the subject clicks a mouse button (or the total time of the trial had passed), and after the post-trial reports from the subject are given (such as the PAS), the command window will again present the writing “Trial ready. Press ENTER to start.”. You may then hide the target stimulus again, and replace it with the next one. Then, repeat the previous step.
8. After the experiment ends, the log file for the experiment can be found under the “Logs” subfolder. The log file is a .mat file, containing two variables: “fields”, holding the names of the columns, and “values”, holding the data from the experiment. Each row in “values” is a single trial. Log files adhere to the naming convention: “Sub_[Subject_ID]_[Date]_[Time]_ExperimentResults.mat”, with the variables in brackets replaced with the relevant values.

### Troubleshooting

**Problem**: After inserting subject details, MATLAB may throw an error, with the top of the stack saying:

Error using **Screen**

See error message printed above.

Error in **PsychImaging** (line 2077)

[win, winRect] = Screen('OpenWindow', screenid, clearcolor, winRect, pixelSize, numbuffers, stereomode, multiSample, imagingMode, specialFlags, clientRect, fbOverrideRect);

**Solution**: Re-run the experiment. If the same problem persists, try restarting MATLAB.

## The parameters file

Many features of the experiment may be altered easily by the parameters file, a simple XLSX file, without having to mess with the code. You can see some parameter files under the “Stim” subfolder of the script.

The parameters file contains several sheets:

1. “Folders” - denoting the relative path to folders which are important for the script: where to keep the log files, where are the stimuli, etc
2. **“Params”** - holding most of the experiment’s parameters, each in a separate line
3. “SubjData” - specifying the subject’s details that need to be provided by the experimenter at the beginning of the experiment
4. “Stimuli” - a list of the stimuli used in the experiment
5. “LogPerTrial” - specifying the information that is logged for each trial

Of these sheets, **“Params”** is the only one that you should alter if you don’t intend to alter the code as well.

### The “Params” sheet

The “params” sheet contains many variables that control the appearance and behavior of the experiment. Each line contains one variable, with the columns describing its name, value, type, “external name” (A somewhat more readable version of its name), and a short description. Following are descriptions of some important variables:

#### Properties of the CFS sequence

- **screenNum**: A variable telling Psychtoolbox where to draw graphics. If you have a “ghost” display connected (see above), choose 2. If you are working with one display only, choose 0. Note that if you have 2 displays connected, you have to choose either “1” or “2”, as choosing “0” will try and draw graphics on both displays as one single big screen, which will lead to unwanted results.
- **mondRate** (“Mondrians_Rate”): in Hz, the rate at which the CFS masks, or “Mondrians”, are changing. Default is 10 Hz.
- **mondCols** (“Mondrians_Colors”): a matrix of RGB values describing the palette of colors that are used for the mondrian shapes. All RGB values are on a scale of [0-1], each line is a color.
- **mondMinSize**\**mondMaxSize**: in pixels, the minimum\maximum size of each shape in the mask, respectively. Size of each shape will be randomly set within this range.
- **cfsOverlayStartContrast**: During a trial, the non-dominant eye sees a 100% white background (turning the goggles completely opaque) which gradually becomes totally black (turning the goggles completely transparent). However, the background doesn’t have to start at 100% opaqueness. This variable, on a scale of [0-1], sets the starting point of opaqueness for the trial.
- **cfsOverlayStartContrast**: Similarly to the previous property, this variable sets the endpoint of opaqueness. That is, the view don’t have to become 100% transparent in the non-dominant eye. This is useful for when you cannot control the light in the room, but still want to keep a low contrast of the target stimulus.
- **cfsOverlayFadeStart** (“CFS_Overlay_Fade_Start”): In ms, how long to wait after the Mondrians start flashing, before starting to diminish the opaqueness of the display in the non-dominant eye (from white to black)
- **cfsOverlayFadeDur**: In ms, how long should the “ramping down” of the opaqueness in the non-dominant eye take.
- **cfsDur**: In ms, for how long should flashing Mondrians be presented. This duration includes the time the script waits until the “ramping down” of opaqueness in the non-dominant eye, and the duration of the “ramping down” itself.
- **occludeVisionAfterTrial**: A boolean. If set to “true”, once the subject reports seeing the target stimulus, or the maximum time for suppression (“cfsDur”) had passed, the goggles become 100% opaque, to block the subject’s view of the real world. Otherwise, the goggles become transparent. Note that the subject may still provide an answer during the span of occlusion. This is useful for short presentation times, like in Experiment 1, where you want to give the subject an option to guess the location of the target stimulus, if they hadn’t seen it during the trial itself.

#### Properties controlling experiment flow

- **eachStimRep** (“Each_Stimulus_Repeats”): The number of times each stimulus should repeat during the experiment, in each level of each condition. In essence, this property sets the number of trials in the experiment.
- **cond_<i>_name, cond_<i>_levels, cond_<i>_lvlsProportions**: For each condition, its name, its possible levels, and the proportions in which these levels should occur (use a vector of “1”’s if you want equal proportions).
- **numConds**: The number of conditions in the experiment
- **constraint_<i>_conds, constraint_<i>_maxReps**: The script allows for the definition of simple constraints on the trials list. These constraints prohibit the repetition of condition levels in consecutive trials. The first variable describes which conditions the constraint is taking into account, and the second variable defines the maximum number of consecutive trials that are allowed to be with the same levels in these conditions. For example, if constraint_1_conds = [1,2], and constraint_1_maxReps = 5, then no more than five consecutive trials with the same level in condition 1 and the same level in condition 2 may appear in the trial list.
- **numConstraints**: The number of constraints on the trial list.
- **showPAS**: A boolean. If set to “1”, shows a Perceptual Awareness Scale (PAS, 1-4) after the suppression is over.
- **PASmaxDuration**: In seconds, if a PAS is shown, for how long should it appear until the subject responds before disappearing. 0=until subject response.

## Basic introduction to the code

The script is written in an OOP approach, and with an attempt at a Model-Presenter-View design pattern. However, it may differ from the classic implementation of this pattern.

All the classes for this experiment are inside the subfolder “\+uri_classes”. In the “\+uri_classes\+common” subfolder you will find classes that define an experiment in general. These in turn are inherited by the classes which define the real-life CFS experiment, found in the subfolder “\+uri_classes\+CFS_MOVERIO”.

The only class instantiated by the script “RunExp_RealLife_CFS.m” is “uri_classes. CFS_MOVERIO.Experiment_Presenter_CFS”. This is the presenter class, which controls the flow of the experiment. The Presenter holds instances of two important classes:
uri_classes.CFS_MOVERIO.Experiment_View_CFS - A class managing the display and interaction with the subject, including controlling of external devices (such as our “puppet theater” device).
uri_classes.CFS_MOVERIO.Experiment_Model_CFS - A class managing data logging and reading. This class is also used to interface with the file system, loading and saving both log files and experiment properties files.
